# NGO-provided free HIV treatment and services in Burkina Faso: scarcity, therapeutic rationality and unfair process

**DOI:** 10.1186/1475-9276-11-11

**Published:** 2012-03-06

**Authors:** Valéry Ridde, Paul Andre Somé, Catherine M Pirkle

**Affiliations:** 1 Research Centre of the University of Montreal Hospital Centre (CRCHUM), 3875 rue Saint-Urbain, Montréal, Québec, H2W 1V1, Canada; 2 Department of Social and Preventive Medicine, University of Montreal, Montreal, Canada; 3 Institut de recherche des sciences de la santé (IRSS) du CNRST, Ouagadougou, Burkina Faso; 4 Independent consultant, Ouagadougou, Burkina Faso; 5 School of Public Health, University of Montreal, Montreal, Canada

**Keywords:** Antiretrovirals, User-fees, Selection processes, Access, West Africa

## Abstract

Until 2010, Burkina Faso was an exception to the international trend of abolishing user fees for antiretroviral treatment (ART). Patients were still expected to pay 1,500F CFA (2 Euros) per month for ART. Nevertheless, many non-governmental organizations (NGOs) exempted patients from payment. The objective of this study was to investigate how NGOs selected the beneficiaries of payment exemptions for government-provided ART and rationed out complementary medical and psychosocial services.

For this qualitative study, we conducted 13 individual interviews and three focus group discussions (n = 13 persons) with program staff in nine NGOs (4,000 patients), two NGO coordinating structures and one national program. These encounters were recorded and transcribed, and their content was thematically analyzed. The results were presented to the NGOs for feedback.

Results indicate that there are no concrete guidelines for identifying patients warranting payment exemptions. Formerly, ART was scarce in Burkina Faso and the primary criterion for treatment selection was clinical. Our results suggest that this scarcity, mediated by an approach we call sociotherapeutic rationality (i.e. maximization of clinical success), may have led to inequities in the provision of free ART. This approach may be detrimental to assuring equity since the most impoverished lack resources to pay for services that maximize clinical success (e.g. viral load) that would increase their chances of being selected for treatment. However, once selected into treatment, attempts were made to ration-out complementary services more equitably.

This study demonstrates the risks entailed by medication scarcity, which presents NGOs and health professionals with impossible choices that run counter to the philosophy of equity in access to treatment. Amid growing concerns of an international funding retreat for ART, it is important to learn from the past in order to better manage the potentially inequitable consequences of ART scarcity.

## Introduction

Like the direct payment of healthcare services in Africa [1], financial access to antiretroviral treatment (ART) has been the subject of numerous debates and considerable research [2,3]. A review of the impact of user fees on HIV/AIDS service delivery demonstrated that abolishing user fees improved equity, by extending access to the poorest users, and clinical results, by facilitating treatment uptake and adherence [2]. Additionally, other studies focused on Africa, such as one conducted in Ethiopia, showed that free ART accelerated a reduction in HIV-related mortality [4]. Studies in Senegal have shown that the absence of fees for ART encouraged treatment adherence [5]. The results of these studies, as well as WHO and UNAIDS recommendations, may explain why the majority of African countries have eliminated user fees for ART [6].

Until 2010, Burkina Faso was an exception to this trend of abolishing fees for ART, despite evidence demonstrating that treatment costs and the costs of complementary services such as laboratory monitoring are important barriers to care [7]. This was somewhat expected for Burkina Faso, as policy-makers have been relatively reticent about all forms of payment exemption. Since the generalization of user-fees in the 1990s, few exemption measures have been put in place and those concerning the most impoverished were never implemented [8]. Only in the past 10 years have a handful of services been made free of charge, such as prenatal consultations and tuberculoses treatment. Thus, the norm in Burkina Faso is that patients pay for care at the point of service delivery. Even when the State occasionally consents to strongly subsidize a program, such as artemisinin-based combination therapies (ACT) and delivery care, it continues to resist completely removing user-fees. This is the case despite the African Union calling for free delivery care and under-five medical consultations [9].

Before 2010, patients were still expected to pay a fee of 1,500F CFA (2 Euros) per month for ART, even though 46% of the population lives on less than 8,400F CFA per month (13 Euros) [10]. While the fee for ART decreased over time (it was 5,000F CFA in 2005), national authorities continued to insist on obtaining user fees from patients on treatment [11,12]. The State and non-governmental organizations (NGOs) did, however, recognize that certain individuals could not pay for treatment and allowed these individuals to receive exemptions.

The medical management of people living with HIV/AIDS (PLWHA) is not limited to the provision of ART. Free antiretrovirals (ARVs) do not translate into free treatment for patients, as there are other financial barriers related to treatment and care. Medications for opportunistic infections, medical exams, mental health care, as well as nutritional support, travel costs, and accommodation services are all a part of the treatment and care "package." Likewise, they can all influence treatment adherence and, consequently, treatment success [13,14]. In Senegal, the direct costs associated with the medical management of PLWHA (not including ARVs) were estimated to be around 15,000F CFA (23 Euros) per month [3]. In Burkina Faso, a 2006 study showed that patients paid a median of 7,000F CFA (11 Euros) per month for medical exams, medications for opportunistic infections and transportation [15].

For historical reasons, in Burkina Faso most care for PLWHA occurs within nationally-based NGOs [16,17]. The State procures ARVs via the Global Fund to Fight AIDS, Tuberculosis and Malaria and distributes them to public services centres or NGOs based on estimated needs. A survey in 2008 showed that approximately 60% of patients on ART in the country were managed by NGOs [18]. For the most part, these NGOs are charities run by PLWHA that offer a number of services besides treatment to support their members. There are financial costs to the patient for each of these complementary services.

Despite the national authorities' insistence on imposing fees for ART in Burkina Faso, a recent study showed that 80% of patients sampled did not pay for treatment [12]. Yet, we do not know how patients were selected for payment exemptions or how other services were rationed out [12]. More importantly, this study was a cross-sectional analysis of those already receiving treatment and thus, did not consider those patients who failed to initiate treatment. One study in Uganda documented that 21% of ART eligible patients were lost to follow-up [19] and in another study in Kenya, 37% of ART ineligible patients were lost to follow-up before becoming ART eligible [20]. Why these patients are lost to follow-up is not known [21], but financial and social barriers have been suggested [19].

The objective of this study is to shed light upon how NGOs selected the beneficiaries of payment exemptions for ART and how they rationed out complementary services. We wanted to know how the ART selection process evolved over time, as ART became increasingly accessible in Burkina Faso. Given that public health programs often neglect the needs of the most impoverished and the most vulnerable [22], the issue of equity is central to our analysis. That is, we wanted to know how patients were targeted for payment exemptions and whether this was fair.

## Methods

This is a qualitative study, which is a research approach that allows us to document targeting processes and to obtain the actors' perspectives on them [23]. In the context of the phenomenon studied here, in which situations change rapidly--such as the international funding retreat that currently has NGOs worried [24]--this means presenting the actors' experiences and reactions to events from a retrospective perspective.

This study looked at NGOs in Burkina Faso that were involved in supporting and managing PLWHA. We chose to focus on NGOs because of their long history of providing ART in Burkina Faso and because they are more explicit in basing their services on social justice than the public sector. We expected NGOs to be more equitable in their selection of participants for ART giving us an idea of what the best-case scenario was for (in) equitable selection processes in HIV treatment. Given the limited time and resources available, we selected a sample of NGOs that were members of the two largest national networks. Among these NGOs, we retained those that: i) had a substantial program, i.e., one following at least 100 PLWHA; ii) had staff who had been involved in the program for more than five years; and iii) were available during data collection (August and September 2008). The resulting sample consisted of nine NGOs, two NGO coordinating structures and one national program (Table 1). No NGO refused to participate in the study.

**Table 1:** Descriptions of NGOs selected for the study

Community organizations with ARV prescription and patient management centres*			
**ORGANIZATION**	**CHARACTERISTIC FEATURES**	**NUMBER OF BENEFICIARIES**	**PLWHA IN TREATMENT BEING FOLLOWED**

**Association 1**	*One of the first to implement a large-scale ARV treatment program. Very influential at the national level and member of several international networks*.	*2692*	*969*

**Association 2**	*One of the first associations of people living with HIV and claiming this status. Member of several international networks*.	*1075*	*273*

**Association 3**	*Association consisting mostly of women living with HIV. One of the first and largest associations of women. Very influential at the national level and in certain international arenas*.	*1947*	*856*

**Association 4**	*Association of women living with HIV. Groups of beneficiaries of a program to fight AIDS. Transformation of a self-support group into an association for treating patients*.	*605*	*284*

Community organizations providing support services for patients on ARV**			

**ORGANIZATION**	**CHARACTERISTIC FEATURES**	**NUMBER OF BENEFICIARIES**	**PLWHA IN TREATMENT BEING FOLLOWED**

**Association 5**	*Association of youth. A prevention-focused organization that has evolved into an organization providing support services to patients*.	*1486*	*308*

**Association 6**	*A prevention-focused organization that has evolved into an organization providing support services to patients. Operates in the areas of community development and poverty alleviation*.	*816*	*406*

**Association 7**	*Relatively young (2004) association of people living with HIV that provides support services to its members*.	*246*	*205*

**Association 8**	*Relatively young (2005) association of people living with HIV whose members were militant for a long time in other associations. Influential at the national level and member of several international networks*.	*550*	*550*

**Association 9**	*Self-help and solidarity association that carries out development activities and that has incorporated the fight against AIDS into its activities for the past 10 years. Member of an international network*.	*540*	*159*

***Coordinating structures***			

**ORGANIZATION**	**CHARACTERISTIC FEATURES**	**ACTIVE FILE**	**PLWHA IN TREATMENT BEING FOLLOWED**

**Coordination 1**	*Coordinating structure for five NGOs involved in the screening council*.	*N/A*	*N/A*

**Coordination 2**	*Coordinating structure for eight NGOs involved in ARV distribution*.	*N/A*	*N/A*

**ARV distribution program**	*One of the largest ART funding programs in Burkina Faso*.	*N/A*	*N/A*

* These organizations have treatment centres that serve as medical centres authorized to prescribe ARVs

** These organizations manage patients on ART and receive their ARVs from a coordinating structure

The majority of NGOs in the study starting offering services thanks to support provided by French NGOs. Association 1 is different from the other associations because of the amplitude and diversity of its financing. At the time of data collection, all of the associations benefited from national financing through the *Programme d'appui au monde communautaire *(PAMAC, funded by UNPD). Associations 1, 3, 5, and 6 have national activities as well as smaller more decentralized ones. The other associations are all local in scope. With the exception of association 9, all can be found in the 2 largest cities of Burkina Faso (Ouagadougou and Bobo-Dioulasso).

In these 12 organizations, we conducted 13 individual interviews with coordinators. We also organized three discussion groups, attended by a total of 13 coordinators from three of these NGOs. The selection of participants for the interviews and for the focus-group discussions was conducted based on the participants' specific knowledge of the study subject. We therefore selected association directors, managers, doctors, and counsellors. Data collection was conducted in French by the second author (PS) based on an interview guide devised in collaboration with the first author. The guide was pretested on two individuals working for an association not included in this sample. The interviews covered the distribution of ART, the selection of beneficiaries, the organization of patients' non-medical services, and problems related to targeting beneficiaries of services provided by the NGOs. Individual interviews lasted between one and one and a half hours. All interviews were digitally recorded and fully transcribed by research assistants in Burkina Faso. The analysis was inductive, without an analytic frame, in order to rest as close as possible to the data. We manually analyzed the content thematically [25]. One of the authors of this article (PS) had been working since 2000 for a national HIV/AIDS NGO (this NGO was not included in the sample and the author has no conflicts of interest with the other NGOs). While we could not conduct participant observation, this "insider knowledge" helped to assure a good contextualization of the data as well as a critical perspective on the participants' discourse. We presented the results to the NGOs in February of 2009 and at the 5th Francophone Conference on HIV/AIDS (Casablanca, Morocco) in March of 2010. The final research report was sent to the participating NGOs.

## Results

### The rejection of user fees and ART beneficiary selection

From the outset, we should note that most of our respondents felt that selecting people to be exempted from payment for services made no sense. For members of these NGOs, this type of targeting was inconceivable. They gave several reasons for their position.

First, even though ART is currently widely available in Burkina Faso, people remembered the 1990s, when ARVs were scarce and necessarily rationed. At that time, "*selecting the beneficiaries of these treatments was hellish*," recalled one program coordinator, who had received 16 treatments thanks to Northern partners. Second, our respondents sometimes invoked the "pay it forward" principle, i.e., since the NGOs received ARVs at no cost, they felt the PLWHA should not have to pay either. Given that these NGOs had provided treatment for free since the beginning, one coordinator said, they "*could not see how they could backtrack now*". Even though they knew "*some people can afford to pay for treatment*", this coordinator continued, it was the idea of payment itself that was inconceivable. "*[Clearly,] we do not agree with this national policy*," another coordinator said. The final reason mentioned was the simple fact of being an NGO member and asking for ART was enough to qualify someone as vulnerable and entitled to receive medications for free.

Outside of this general view, there were a few individuals who supported patients' financial participation in accessing ART. They adopted this counter-position from the perspective of preventing scarcity. Some believed that financial contributions from patients able to pay the treatment fee would help buffer against the threat of recurrent stock-outs.

### Selection criteria in situations of scarcity

When treatments first became available, most NGOs had only a few units of ARVs and rationing was obligatory [14]. The first criterion for selecting beneficiaries was clinical, related to the conditionalities of treatment management. Thus, "*if we had five treatments for patients, those who had had their full clinical work-up [CD4, viral load] were first in line*," one physician told us. In this case, people's ability to pay for this costly workup was a factor in their being selected. However, he later explained that some people who had not had the workup, but whose clinical condition was obvious enough to justify treatment, were also treated. Given the importance of adherence to treatment, NGOs also applied criteria other than medical indications alone. One NGO physician explained that having a companion to support treatment adherence was an important criterion. Thus, people who were socially isolated were excluded until they could find a companion. Likewise, he added, another factor considered in patient selection was a history of good compliance with antibiotic treatment (e.g. Cotrimoxazole, used to prevent opportunistic infections before the arrival of ARVs).

The previous scarcity of ARVs created a situation in which providers selected patients based who was most likely to succeed on treatment. One program coordinator told us, "*it was difficult for us to decide to put someone on a treatment that was very costly back then, with no assurance that this person would follow the regimen as diligently as was required*." Thus, as this coordinator explained, even though the NGO sometimes gave ART to "*the really poor... this was wasted, because they didn't take their medications as indicated*," particularly because the poor were often isolated and without "*companions*" to help them follow the treatment. Finally, with respect to ART activism, a coordinator told us, "*in this NGO, seniority and militancy were the criteria applied*." The coordinator explained that, for the NGO to survive and continue to fight for its cause, its most militant activists needed to stay alive; "*without ART, they were in danger of being lost*," so they were the first beneficiaries.

### Triage for complementary services

Besides the provision of ARVs, HIV/AIDS treatment includes additional services that are both medical (treatments for co-morbidities, laboratory tests, etc.) and social (nutritional and financial support, assistance to orphans and vulnerable children). None of these services are free, and decisions must be made as to who will receive them. In providing nutritional support, schooling for orphaned children, or even financial support, the NGOs insisted that their concern has always been to serve the most impoverished first. One program coordinator told us, "*if someone is well-off socio-economically, he will receive fewer benefits than someone who seems to be at a lower level*." This selection was based on the regularity of patients' incomes, their type of work, or their "*standing*" as assessed through home visits. Widows were also among the categories directly targeted by NGOs. Aside from these broad categories, none of the NGOs had any more precise criteria or more discriminating indicators.

However, most of our respondents thought that self-selection, one form of targeting [26], was widely practised. Our respondents believed that those using NGOs were mostly people who were destitute, often victims of discrimination; "*someone who has three meals a day wouldn't come and stand in a long line-up for a few kilograms of flour*."

Beyond these statements, no one was able to supply us with any evidence that these criteria actually resulted in complementary services being accessed by the worst-off. Ultimately, those who received such services had already passed through the first filter (i.e., they had already been selected to receive ART); hence, it is unlikely that they were the worst-off.

### Criteria are loosely defined and applied with uncertainty

The NGOs did not have any official process to define their selection criteria. We found no documentation on this subject and had to rely only on our respondents' statements. Sometimes an NGO would turn to the services of the Ministry for Social Action, who is responsible for issuing indigence certificates. However, "*they said that, even on their end, they couldn't keep up and were unable to produce indigence certificates because it took time and resources*," one program coordinator recalled. However, sometimes the contrary situation arose, in which an agent of the Ministry would refer a patient to the NGO because the NGOs were better acquainted with indigents in the community. In some hospitals, in the absence of indigence certificates, which were supposed to be the only acceptable official document, the medical team would organize its own patient selection committee. However, this was a burdensome responsibility, "*doctors were often left to their own conscience, and that's precisely why we said, if only we could declare everything to be free, once and for all...*" Only one of the NGOs set up a selection committee, but "*this presented an ethical dilemma because we didn't have a clear and distinct methodology*."

This lack of any official definition was reflected in a rather arbitrary application of selection criteria by people without training for such selection processes. NGO staff were left on their own in applying the criteria to the people they encountered, either at the NGO or during home visits. Agents' subjective assessments, as well as their experience, came into play; "*we're in contact with each other, we know who is who*," one coordinator said. Others worried about targeting errors, because "*when a man is faced with resources, he can change radically and tell you things that are not necessarily true, just to benefit from those resources*."

## Discussion

### The consequences of ARV scarcity & sociotherapeutic rationality

At the national level, at the time of this writing, there were no apparent stock-outs of State-procured ARVs. Ministry officials told us that there seemed to have been no stock-outs since mid-2007. However, because the country was among those, in 2008, who reported still experiencing supply problems [27], we cannot conclude that at present, there are no problems in the distribution chain. On the continent as a whole, there is concern that supply-chain disruptions will increase as funders cap, reduce, or withdraw funding from ART programs [24,28].

The past high cost of ART coupled with sporadic stock-outs led to a situation of scarcity that the actors in our study recalled all too well. Therefore, the results of this study suggest that scarcity, mediated by sociotherapeutic rationality, may have led to a situation of inequities in the provision of ART. We use the term sociotherapeutic rationality to describe the decision-making process that ART providers use in choosing beneficiaries of treatment. This process weighs social facilitators and risk factors (e.g. lack companion for adherence assistance, poverty, etc.) with potential patient outcomes. The goal of this rationality is to maximize clinical success and minimize the chances that patients "waste" scarce treatment and services.

The first explicit criterion in this sociotherapeutic rationality was clinical success. Those deemed most likely to succeed on treatment were able to obtain the necessary laboratory workup (CD4 count, viral load) and had often shown previous ability to adhere to medications such as Cotrimoxazole. The most socially and economically marginal members of society were, and continue to be, those least likely to overcome these barriers and consequently, many are lost to follow-up prior to becoming eligible for treatment [19,20]. There is little research describing the socioeconomic characteristics of those lost to follow-up prior to ART initiation. One study showed that ART ineligible patients lost to follow-up had significantly lower BMIs than those who started treatment, suggesting poorer nutritional status [20]. We also know that after starting ART, loss to follow-up is associated with the inability to pay for transport to health centres, as well as work and childcare responsibilities [29] and that those returning very late for treatment (CD4 less than 100) are more likely to be women with no education and unmarried men [30]. Unlike patients already on ART, who receive medications and feel physical improvements with treatment, ART ineligible patients receive few such incentives and often have to pay for monitoring services [20]. Those unable or unwilling to do so, will be lost before being selected into treatment. Some will return with advanced AIDS while a substantial minority will die. In South Africa and Zambia, community support has been shown to improve ART success and program retention [31,32]. However, in Burkina Faso, this type of community support for PLWHA is limited and is largely a role taken on by NGOs and rarely the public health system. Here, NGOs help provide nutritional support, education on hygiene, and adherence support.

Social criteria, such as having a companion to help with treatment adherence, were also a part of the sociotherapeutic rationality used to select beneficiaries of treatment. Those without means, such as orphans and widows, were either excluded or forced to delay treatment until a companion could be found. This is a situation similarly described by Vuarin [33] in the neighboring country of Mali, where people with means had greater social networks and support.

Delayed initiation of ART is a well-known risk factor for early mortality on treatment [34,35]. More recently, high one-year mortality has been demonstrated in those who were ART eligible but failed to initiate treatment [19]. Socioeconomic barriers that cause delays in receiving treatment are, for many, equivalent to being excluded because of the strong association between delay and death.

NGOs and doctors employing this sociotherapeutic rationality find themselves in a tragic dilemma that prevents them from being able to care for the sick without discrimination. Sociotherapeutic rationality is constructed to the detriment of equity (it is unfair), since the most impoverished lack the resources to pay for services, such as viral load (often conducted in Europe or Côte d'Ivoire), that would increase their chances of being selected for treatment.

The idea of sociotherapeutic rationality invokes related work in West Africa on therapeutic citizenship [36]. Nguyen's research showed that claims were made on the global social order based on a therapeutic predicament (e.g. being HIV positive). At the beginning of the period of access to ART, in order to make the strongest claim on that social order, it was necessary to triage among the most charismatic individuals to demonstrate publicly and internationally that adherence to treatment in Africa was possible [14]. It was vital to prove clinical success to increase the flow of ARVs and this was best done by selecting individuals most likely to succeed on treatment (those with the support to be adherent to treatment and the finances to pay for clinical monitoring). While treatment has become more accessible, the vestiges of this history of scarcity remain.

### Rationing-out of complementary treatment services

The provision of complementary services, such as nutritional and financial support, is likewise geared towards the patient's ultimate success. These complementary services are important elements of the sociotherapeutic rationality that NGOs employ in selecting service beneficiaries. For example, it is well known that food insecurity compromises ART adherence and reduces the clinical effectiveness of treatment [37]. Similarly, financial support to help patients pay for transportation to medical appointments or laboratory testing is intended to improve medical management and, thus, chances of treatment success. None of these services are free of charge to patients in Burkina Faso [15], and NGOs must triage patients to determine who should benefit from these services. The results of this study reveal that there are no concrete guidelines for identifying beneficiaries, a situation that could intensify inequities, such that some patients would receive adequate care and others, care that is substandard. It is important to note that there are no comprehensive food or financial aid programs supported by the NGOs in this study, which may explain why people selected into ART programs benefit disproportionately from complementary services.

On the other hand, it should be noted that, once the issue of ART eligibility had been resolved, NGOs tried to restore relative equity in the selection of beneficiaries for complementary services. Still, we can hypothesize that, for as long as the criteria remain arbitrary, initial self-selection into the programs will mean that the chief beneficiaries of such services will be the least poor among the worst-off, i.e., those who can afford the prerequisite workup [26]. This still needs to be demonstrated.

### Abolishing fees for ART: insufficient to ensuring equity

The NGOs and the government of Burkina Faso have inherently divergent perspectives on patients' financial participation in ART. For mostly social justice reasons, NGOs in the country are fundamentally against fees for treatment, a position supported by WHO and UNAIDS [6]. At the same time, despite previous government policy, some government-run public health facilities also provided free ART [12]. There was thus a disconnect between political discourse and the actual situation on the ground. Indeed, the government's official position is that it has always been preferable that patients pay for treatment, as was demonstrated in an official speech during the organization of a conference on ART toward the end of 2008 [38]. One reason invoked for payment and supported by a few NGOs was concern for the sustainability of treatment access. Yet treatment is largely funded by international donors and not the State. Further, the monthly treatment fee requested by the State does not fully recover the cost of treatment. One study in Senegal showed, for instance, that the government had the financial means to treat patients without requiring user fees [39].

Given increasing evidence that eliminating fees for ART increases treatment adherence, rendering ART free in Burkina Faso could, in fact, improve long-term sustainability. This is because population-level improvements in treatment adherence could slow the development of drug resistance [40], an issue that is all the more salient given primary resistance levels already documented to be as high as 29% in certain West African settings like Mali [41] and at 13% in Ouagadougou Burkina Faso [42]. Drug resistance, of course, necessitates the use of more expensive second- and third-line treatments, threatening the long-term sustainability of national ART programs.

The counter-position taken by a minority of our participants in favour of user fees mirrored that of the State, in that it reflected a concern about treatment scarcity. Specifically, certain participants believed that user fees could buffer against stock-outs. Stock-outs of ARVs were a reality in some health facilities in Burkina Faso in 2007 [43], as elsewhere on the continent [6,27]. One the biggest challenges to scaling-up treatment access is the prerequisite reinforcement of fragile and complex supply chains. Stock-outs also reduce patient motivation to adhere to treatment [13]. Once again, the underlying logic behind this counter-position in favour of user fees is to maximize treatment efficacy; but this time, instead of focusing on those individuals most likely to succeed on treatment, the focus is on reducing scarcity in the first place.

With the exception of some discussion around the provision of complementary services to widows, it was rare for participants to consider equity explicitly in the selection of beneficiaries. This lack of concern for the least well-off is not new; health policy decision-makers in Burkina Faso have often focused on equality of access to the detriment of equity [8]. The nearly ubiquitous focus on maximizing treatment success may stem from the initial battle to obtain ARVs in developing countries. Advocates for treatment access in developing countries had to "prove" that clinical outcomes in resource-poor countries could be as good as in resource-rich countries and that Northern partners would not be "wasting" money by supplying treatment to these contexts [14].

### Study limitations

This study was conducted just prior to the 2010 decision by the Burkina Faso government to eliminate user-fees for ART. Thus, results from this study should be understood from a retrospective perspective. It will be interesting to follow how NGOs adapt to the new situation. The results of this article should not be generalized as applying to the entire historical situation of ART access in Burkina Faso as we did not interview participants from the public sector nor did we meet with representatives of every NGO in the country. Further, the number of participants we met in each NGO was relatively limited. Finally, this study is based uniquely on qualitative data and should be understood to represent the discourse and interpretations of the informants we interviewed.

## Conclusion

The provision of ART in Burkina Faso has changed dramatically in the past two years. At the time of data collection, there were still user-fees for ART. On December 31, 2009, after years of struggle, the NGOs of Burkina Faso and many health professionals and researchers scored a major victory. The President of the Republic announced that ART would be free in Burkina Faso. While this calls for celebration, some caution is also in order. In removing the financial barrier to access, the problem of selecting patients who will receive treatment is resolved, if they are not lost prior to becoming ART eligible. However, this decision will only be equitable to the extent that it is actually applied, that stock-outs do not recur, and that resources are allocated to ensure that complementary services (particularly laboratory monitoring and transportation) and social support will be accessible to patients unable to pay. If these conditions are not met, it is possible that inequitable allocation of treatment and complementary services will reoccur.

Concerns about repeating previous injustices are not without merit. There is growing concern of an international funding retreat for ART. The worldwide financial crisis, donor fatigue, and the fraudulent use of Global Fund money has encouraged donors to cap, reduce, or withdraw funds from HIV treatment programs [24,28]. The global funding retreat has led to concerns about increased treatment stock-outs, inability to add new patients to treatment programs, and the re-emergence of ARV user-fees [28,44]. In light of this, it is all the more important to learn from the past in order to better manage the "unfair" consequences of ART scarcity.

## Competing interests

The authors declare that they have no competing interests.

## Authors' contributions

VR and PAS wrote the research protocol. PAS coordinate the qualitative data collection and analysis with VR. VR and CP wrote the first draft. All authors read, improved and approved the final manuscript.

## References

[B1] Ridde V Morestin F A scoping review of the literature on the abolition of user fees in heathcare services in Africa Health Policy Plan 2010 26 1 1 11 20547653 10.1093/heapol/czq021

[B2] Souteyrand YP Collard V Moatti JP Grubb I Guerma T Free care at the point of service delivery: a key component for reaching universal access to HIV/AIDS treatment in developing countries AIDS 2008 22 Suppl. 1 S161 S168 18664948 10.1097/01.aids.0000327637.59672.02

[B3] Taverne B Gratuité des traitements du sida en Afrique: un impératif de santé publique Population, développement et VIH/Sida et leurs rapports avec la pauvreté 2005 ONU. New York: Report presented at the 38th Session of the UN Commission on Population and Development April 4-8, 200522396489

[B4] Reniers G Araya T Davey G Nagelkerke N Berhane Y Coutinho R Sanders EJ Steep declines in population-level AIDS mortality following the introduction of antiretroviral therapy in Addis Ababa, Ethiopia AIDS 2009 23 511 518 10.1097/QAD.0b013e32832403d0 19169138 PMC2666986

[B5] Laniece I Ciss M Desclaux A Diop K Mbodj F Ndiaye B Ndoye I Adherence to HAART and its principal determinants in a cohort of Senegalese adults AIDS 2003 17 Suppl. 3 S103 S108 14565616 10.1097/00002030-200317003-00014

[B6] Gilks CF Crowley S Ekpini R Gove S Perriens J Souteyrand Y De Kock K The WHO public-health approach to antiretroviral treatment against HIV in resource-limited settings Lancet 2006 368 9534 505 510 10.1016/S0140-6736(06)69158-7 16890837

[B7] Nguyen V-K Grennan T Peschard K Tan D Tiendrébéogo I Antiretroviral use in Ouagadougou, Burkina Faso AIDS 2003 17 Suppl. 3 S109 S111 14565617 10.1097/00002030-200317003-00015

[B8] Ridde V "The problem of the worst-off is dealt with after all other issues": the equity and health policy implementation gap in Burkina Faso Soc Sci Med 2008 66 1368 1378 10.1016/j.socscimed.2007.10.026 18248864

[B9] African Union Assembly of the African Union 2010 Kampala, Uganda: Fifteenth Ordinary Session 25 27 July 2010

[B10] INSD Annuaire statistique. Édition 2008 2009 INSD: Ouagadougou

[B11] Foro A Ridde V Analyse comparée des plans stratégiques de lutte contre le VIH en Haïti et au Burkina Faso Science et technique, Sciences de la santé, Spécial hors série n°1 2008 75 84

[B12] Kouanda S Bocoum Yaya F Doulougou B Bila B Yaméogo M Sanou M Sawadogo M Sondo B Msellati P Desclaux A Obermeyer C User fees and access to ARV treatment for persons living with HIV/AIDS: implementation and challenges in Burkina Faso, a limited-resources country AIDS Care 2010 22 9 1146 52 10.1080/09540121003605047 20824567

[B13] Merten S Kenter E McKenzie O Musheke M Ntalasha H Martin-Hilber A Patient-reported barriers and drivers of adherence to antiretrovirals in sub-Saharan Africa: a meta-ethnography Trop Med Int Health 2010 15 16 33 20586957 10.1111/j.1365-3156.2010.02510.x

[B14] Nguyen V-K Ako CY Niamba P Sylla A Tiendrébéogo I Adherence as therapeutic citizenship: impact of the history of access to antiretroviral drugs on adherence to treatment AIDS 2007 21 Suppl. 5 S31 S35 18090265 10.1097/01.aids.0000298100.48990.58

[B15] Bocoum Yaya F La prise en charge des patients infectés par le VIH/SIDA au Burkina Faso: une revue des coûts supportés par les patients. Paper presented at the Symposium sur L'accès aux traitements antirétroviraux: leçons des expériences 2008 Burkina Faso: Ouagadougou

[B16] Desclaux A Kouanda S Obermeyer CM Stakeholders' participation in operational research on HIV care: insights from Burkina Faso AIDS 2010 24 Suppl. 1 S79 S85 20023444 10.1097/01.aids.0000366086.21687.52

[B17] Somé P-A Ridde V La place de la participation dans les processus évaluatifs des politiques de lutte contre le VIH au Burkina Faso Science et technique, Sciences de la santé, Spécial hors série n° 1 2008 65 73

[B18] CNLS Bilan général de la mise en oeuvre du plan national multisectoriel de lutte contre le VIH et les IST (PNM) de l'année 2008 2009 Ouagadougou: CNLS: VIIIème session ordinaire du CNLS-IST

[B19] Geng E Muyindike W Glidden D Bwana M Yiannoutsos C Braitstein P Musinguzi N Bangsberg D Martin J EA-IeDEA Failure to initiate ART, loss to follow-up and mortality among HIV-infected patients during the pre-ART period in Uganda: understanding engagement in care in resource-limited settings 2011 Boston: Conference on Retroviruses and Opportunistic Infections

[B20] Kohler P Free CTX substantially improves retention among ART ineligible clients in a Kenyan HIV treatment program 2011 Boston: Conference on Retroviruses and Opportunistic Infections

[B21] Smart T Adherence and retention in HIV care in resource limited settings 2011 Aidsmap, editor. London

[B22] Victora CG Wagstaff A Schellenberg JA Gwatkin D Claeson M Habicht JP Applying an equity lens to child health and mortality: more of the same is not enough Lancet 2003 362 9379 233 241 10.1016/S0140-6736(03)13917-7 12885488

[B23] Patton MQ Qualitative research and evaluation methods 2002 3 Thousand Oaks, London, New Delhi: Sage Publications

[B24] Médecins Sans Frontières No time to quit: HIV/AIDS treatment gap widening in Africa 2010 Brussels: Médecins Sans Frontières

[B25] Paillé P Mucchielli A L'analyse qualitative en sciences humaines et sociales 2003 Paris: Colin22396904

[B26] Hanson K Worrall E Wiseman V Mills A, Bennett S, Gilson L Targeting services towards the poor: a review of targeting mechanisms and their effectiveness Health, economic development and household poverty: from understanding to action 2007 London: Routledge 134 154

[B27] WHO Towards universal access: scaling up priority HIV/AIDS interventions in the health sector: progress report, September 2009 2009 Geneva: World Health Organization

[B28] Moszynski P Donor fatigue is slashing access to AIDS care in Africa, warns charity BMJ 2010 340 c2844 10.1136/bmj.c2844 20508020

[B29] Geng E Bangsberg D Musinguzi N Emenyonu N Bwana M Yiannoutsos C Understanding reasons for and outcomes of patients lost to follow-up in antiretroviral therapy programs in Africa through a sampling-based approach J Acquir Immune Defic Syndr 2010 53 405 411 10.1097/QAI.0b013e3181b843f0 19745753 PMC3606953

[B30] Pirkle CM Nguyen V-K Ag Aboubacrine S Cissé M Zunzunegui M-V Socio-demographic correlates of late treatment initiation in a cohort of patients starting antiretroviral treatment in Mali, West Africa AIDS Care 2011 23 11 1425 1429 10.1080/09540121.2011.565026 22022850

[B31] Wouters E Van Damme W Van Loon F van Rensburg D Meulemans H Public-sector ART in the Free State Province, South Africa: community support as an important determinant of outcome Soc Sci Med 2009 69 8 1177 1185 10.1016/j.socscimed.2009.07.034 19692165

[B32] Zachariah R Teck R Buhendwa L Fitzerland M Labana S Chinji C Humblet P Harries AD Community support is associated with better antiretroviral treatment outcomes in a resource-limited rural district in Malawi Trans R Soc Trop Med Hyg 2007 101 1 79 84 10.1016/j.trstmh.2006.05.010 16962622

[B33] Vuarin R L'argent et l'entregent Cahier des sciences humaines 1994 30 1-2 255 271

[B34] Bekker LG Egger M Wood R Early antiretroviral therapy mortality in resource-limited settings: what can we do about it? Curr Opin HIV AIDS 2007 2 4 346 351 10.1097/COH.0b013e3281e72cbd 19372910

[B35] Braitstein P Brinkhof MW Dabis F Schechter M Boulle A Miotti P Mortality of HIV-1-infected patients in the first year of antiretroviral therapy: comparison between low-income and high-income countries Lancet 2006 367 9513 817 824 16530575 10.1016/S0140-6736(06)68337-2

[B36] Nguyen V-K Ong A, Collier SJ Antiretroviral globalism, biopolitics, and therapeutic citizenship Global assemblages: technology, politics, and ethics as anthropological problems 2005 Oxford: Blackwell Publishing

[B37] Cantrell RA Sinkala M Megazinni K Lawson-Marriott S Washington S Chi BH Stringer JS A pilot study of food supplementation to improve adherence to antiretroviral therapy among food-insecure adults in Lusaka, Zambia J Acquir Immune Defic Syndr 2008 49 2 190 195 10.1097/QAI.0b013e31818455d2 18769349 PMC3847664

[B38] Kouanda S Accès au traitement antirétroviral: leçons des expériences Science et technique, Sciences de la santé, Spécial hors série n°1 2008 102 116

[B39] Taverne B Diop K Vinard P Coriat B The cost of universal free access for treating HIV/AIDS in low income countries: the case of Senegal The political economy of HIV/AIDS in developing countries: TRIPS, public health systems and free access 2008 London: Edward Elgar 273 290

[B40] Wainberg MA Friedland G Public health implications of antiretroviral therapy and HIV drug resistance JAMA 1998 279 24 1977 1983 10.1001/jama.279.24.1977 9643862

[B41] Haidara A Chamberland A Sylla M Aboubacrine S Cissé M Traore H ATARAO Group High level of primary drug resistance in Mali HIV Med 2010 11 6 404 411 20146734 10.1111/j.1468-1293.2009.00806.x

[B42] Tebit DM Sangaré L Tiba F Saydou Y Makamtse A Somlare H Bado G Kouldiaty BG Zabsonre I Yameogo SL Sathiandee K Drabo JY Krausslich HG Analysis of the diversity of the HIV-1 pol gene and drug resistance associated changes among drug-naive patients in Burkina Faso Medical Virology 2009 81 10 1691 1701 10.1002/jmv.21600 19697403

[B43] Munro K Addressing adherence to antiretroviral therapy in Ouagadougou, Burkina Faso: insights from hospital ethnography (Unpublished master's thesis) 2009 Montreal, Quebec, Canada: University of Montreal

[B44] Anonymous Funding cuts spark fears about the rise of drug-resistant strains of HIV in Africa CMAJ 2010 182 3 E165 E166 10.1503/cmaj.109-3149 20064947 PMC2826492

